# Rapid and precise alignment of raw reads against redundant databases with KMA

**DOI:** 10.1186/s12859-018-2336-6

**Published:** 2018-08-29

**Authors:** Philip T. L. C. Clausen, Frank M. Aarestrup, Ole Lund

**Affiliations:** 1 Department of Bioinformatics, Technical University of Denmark, 2800 Kgs Lyngby, Denmark; 2 Research Group for Genomic Epidemiology, National Food Institute, Technical University of Denmark, 2800 Kgs Lyngby, Denmark

## Abstract

**Background:**

As the cost of sequencing has declined, clinical diagnostics based on next generation sequencing (NGS) have become reality. Diagnostics based on sequencing will require rapid and precise mapping against redundant databases because some of the most important determinants, such as antimicrobial resistance and core genome multilocus sequence typing (MLST) alleles, are highly similar to one another.

In order to facilitate this, a novel mapping method, KMA (*k*-mer alignment), was designed. KMA is able to map raw reads directly against redundant databases, it also scales well for large redundant databases. KMA uses *k*-mer seeding to speed up mapping and the Needleman-Wunsch algorithm to accurately align extensions from *k*-mer seeds. Multi-mapping reads are resolved using a novel sorting scheme (ConClave scheme), ensuring an accurate selection of templates.

**Results:**

The functionality of KMA was compared with SRST2, MGmapper, BWA-MEM, Bowtie2, Minimap2 and Salmon, using both simulated data and a dataset of *Escherichia coli* mapped against resistance genes and core genome MLST alleles. KMA outperforms current methods with respect to both accuracy and speed, while using a comparable amount of memory.

**Conclusion:**

With KMA, it was possible map raw reads directly against redundant databases with high accuracy, speed and memory efficiency.

**Electronic supplementary material:**

The online version of this article (10.1186/s12859-018-2336-6) contains supplementary material, which is available to authorized users.

## Background

In bioinformatics, the oldest and probably the single most important tool is the alignment of one or more sequences. Alignments informs us how similar a sequence is compared with another sequence and can be used to quantify the abundance of any similar sequence pattern. If the found pattern has an annotation, this can be transferred to the query sequence. The proper choice of alignment method is thus critical for the further investigations [1].

Over the past decade several mapping methods have been developed that enable direct mapping of raw reads against target sequences. Previous studies have shown read-mapping based approaches to be superior to the more traditional methods where the reads are first assembled, and the assembly is hereafter BLAST’ed. against a database. The success of such methods are highly dictated by the quality of the assembly, because BLAST remains too slow to map the raw reads directly. Which is problematic within repeat and low depth regions of the genome, where the assembly often results in gaps between the assembled contigs, leading to missing data [2, 3]. Today Bowtie2 [4], BWA-MEM [5] and Minimap2 [6] are popular mapping methods that allows for fast mapping and alignment of raw reads against large reference genomes. All of these methods can be adapted to map against entire databases of sequences, but suffer from random selection when there is a tie for best match. In order to resolve these ties, several tools have been designed to resolve these random selections with extensive pre- and post-processing. Examples of these are SRST2 [3] and MGmapper [7] which both pre- and post-process the sequences for mapping with Bowtie2 and BWA-MEM, respectively. Other methods, such as Salmon [8], uses the EM algorithm in order to estimate the sequencing level of homologous templates from mappings generated by traditional methods.

The redundant databases used in genomic epidemiology are however still posing a challenge regarding mapping raw reads directly. When databases are constantly updated with new sequences due to natural evolution, the results are in a constantly changing state, making clustering difficult. This feature of the databases makes direct mapping of raw reads troublesome, as there is no guarantee that the read will cover a unique part of a reference sequence, resulting in a tie for best match. Redundancy is a problem when mapping to bacterial databases, but to a lesser degree since these databases are not as redundant as some of the gene databases, and better use can be made of paired end reads since only one of the ends will often map to single genes. There is a need for new methods to resolve the issue with redundancy in an accurate and fast manner so that acute decisions can be made. In response to this need, a new alignment method, KMA (*k*-mer alignment), was developed. KMA introduces a novel sorting scheme, ConClave, in order to distinguish homologous templates.

## Methods

**Fig. 1:**
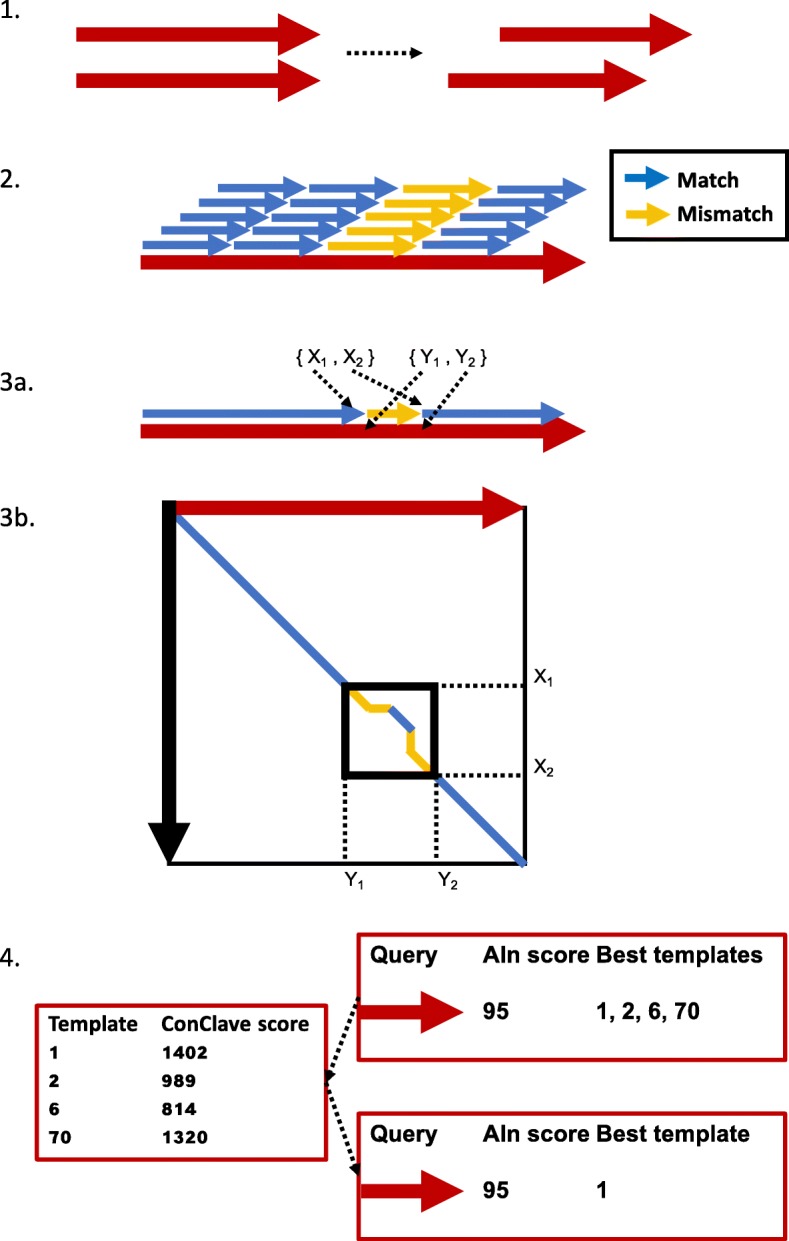
Overview of step 1–4 of the KMA algorithm. 1: trim reads. 2. Match k-mers between query and database. 3a: Extend matching k-mer seeds, and identify regions with mismatches. 3b: Use the Needleman-Wunsch algorithm to align regions of mismatching k-mers. 4: Conclave scoring used to choose one best-aligning template per query sequence.

We introduce a novel alignment method, KMA, and scoring scheme, ConClave, which allows for mapping of raw reads directly against redundant databases. KMA diverges from known mappers by allowing redundancy within the databases, and KMA also produces consensus sequences and a result overview. KMA was created to be as intuitive and user friendly as possible, based on our current user demands and analysis challenges. In order to solve this, KMA works in five main steps: trimming of reads, heuristic *k*-mer mapping, fine alignment, ConClave scoring and reference assembly (see Fig. 1).

### Heuristic *k*-mer mapping

When mapping against databases, a query sequence can fall within two categories: the query does not look like anything in the database or the query looks like something in the database. Depending on the database, it is therefore necessary to determine at an early stage whether alignment of a given sequence is feasible or if the sequence should be discarded from further analysis. Computationally, it is much faster to map raw reads against template sequences and determine their approximate origin than it is to perform alignment giving the exact differences between sequences. In order to exploit this, KMA maps *k*-mers between the query sequence and template database. The matching *k*-mers are then used to produce a mapping score where a matching *k*-mer is rewarded with a score of *k* while *k*-mer mismatches are penalized with − 1. For a query sequence to be accepted for alignment, it must have a positive score based on this mapping, equivalent to a minimum identity of (*k* + 1)^− 1^ in *k*-mer space. In the case where a query sequence is accepted, the potential template candidates are limited by linking the matching *k*-mers to their respective template sequences.

### Fine alignment

KMA uses a hash map of indexed *k*-mers from which to start alignment, where this indexing is computed for templates passing the heuristic *k*-mer mapping. The indexed *k*-mers are then used as seeds from which to start alignment, where each matching seed is extended to give an optimal score. To enable a high resolution of gaps and mismatches, KMA utilizes the Needleman-Wunsch algorithm [9] to align the pieces of query and template sequence between seed-extends. The tails of the alignment, before the first seed and after the last, are aligned with a specialized version of the Needleman-Wunsch algorithm allowing for early stopping so that leading and trailing gaps can be skipped.

### ConClave scoring

As KMA is designed to map sequences against redundant databases, the possibility of a tie for best matching template should be considered more as rule than an exception. For KMA to be able to find the one most likely template for each query sequence, a novel scoring scheme, ConClave, was developed.

The ConClave scoring scheme requires that all best matching templates are initially accepted for all best matching query sequences. The alignment scores obtained are then summed together for each template candidate in order to form the ConClave score, in this way reflecting a maximum score for each template. For alignments where ties do not occur, a second score is conducted as the unique ConClave score, which are used to reflect the unique alignments.

Ties for best matching templates can now be resolved by choosing the most likely template based on the ConClave score. In case of a tie on the ConClave score, the unique ConClave score is used to choose a best matching template. For samples were two templates are equally well-suited candidates, reflected by identical ConClave scores, the algorithm will choose the first sequence added to the database.

### Reference assembly

The ConClave scoring and alignment of reads enables assembly guided by a reference, resulting in a consensus sequence for the given reference / template. From the alignment, the differences between each read and the associated template are known, and this gives information about which nucleotides are called at each position in the template. Each nucleotide is then determined by majority voting, where the strength of the called nucleotide is tested with a McNemar test, giving a robust base calling performance across different sequencing platforms [10]. In case a nucleotide is not significantly overrepresented according to the McNemar test (according to a user specified α, default 0.05), it is reported in lower case.

After reference assembly, statistics for each template are collected in a single file, containing features such as template identity, coverage and depth.

### Performance evaluation and comparison

The performance of KMA was evaluated on a dataset of 13 *Escherichia coli* from Grad et al. (2012). The dataset included 11 *E. coli* from the German / French outbreak in 2011 and two historical *E. coli* [11]. Phenotypic susceptibility tests were known for all of the 13 *E. coli*, and the presence of the beta-lactamase blaCTX-M-15 were verified by PCR [11].

The 13 *E. coli* were mapped to the ResFinder database of known resistance genes [12], where the associated phenotype of each gene were compared with phenotypic susceptibility tests.

In order to measure the performance on larger databases, a core genome MLST (cgMLST) database for *E. coli* was downloaded from EnteroBase (available at: http://enterobase.warwick.ac.uk [Accessed 18 January 2018]. The cgMLST scheme contains 2447741 closely related genes, which together matches the size of the human genome.

KMA was compared with six different methods: SRST2 (v. 0.1.8) [3], MGmapper (v. 2.7) [7], BWA-MEM (v- 0.7.15) [5], Bowtie2 (v. 2.2.4) [4], MiniMap2 (v. 2.6) [6] and Salmon (v. 0.9.1) [8], each capable of mapping raw reads directly against entire databases of sequences.

SRST2 uses Bowtie2 to map against a homology-reduced database, clustered at 90% identity using CD-hit [13]. From each cluster, one allele is chosen, as the one with the highest alignment score from all alignments towards that cluster. After mapping and alignment, SRST2 performs SNP calling and reports the differences between query and template sequences [3].

For MGmapper to accept a template, it has to contain at least one unique mapping read when mapped with BWA-MEM. After mapping the reads, MGmapper reports the depth and template coverage of the accepted genes [7].

Salmon was developed for quantifying RNA transcript levels using the EM-algorithm. Which partially matches the redundant database problem, as only a subset of the templates in the database are likely to be present. Salmon can be used as an extension to BWA-MEM, Bowtie2 and Minimap2, when the alignment method is set to report several alignments per read [8].

SAMtools and BEDTools were used to estimate depth and template coverage of mappings performed with BWA-MEM, Bowtie2 and Minimap2 so that these could be used for a more direct comparison as well [14, 15].

To prove the difficulty of mapping raw reads directly against redundant databases, a simulated dataset of single-end and paired-end reads was created, where each gene in the ResFinder database was split into raw reads with a length 100 bp, and an insert size of 250 for the paired end set. Error and indel rates were set to zero so that the simulated reads would represent the perfect case scenario of short-read sequencers. Meaning, that any inconsistencies from the mapping methods, would reveal each of their weaknesses in mapping against redundant databases, and give a clear view of their assumptions. Simulated reads were created with chopDB, which is included alongside the commands used to compare KMA with existing methods and the ResFinder database.

## Results

Salmon was not used in combination with Minimap2, because Minimap2 did not report more than one alignment per read no matter how the “-N” and “-p” options were set.

### Simulated datasets

For the simulated datasets, the comparison was straight forward as the location of every read was known.

**Fig. 2:**
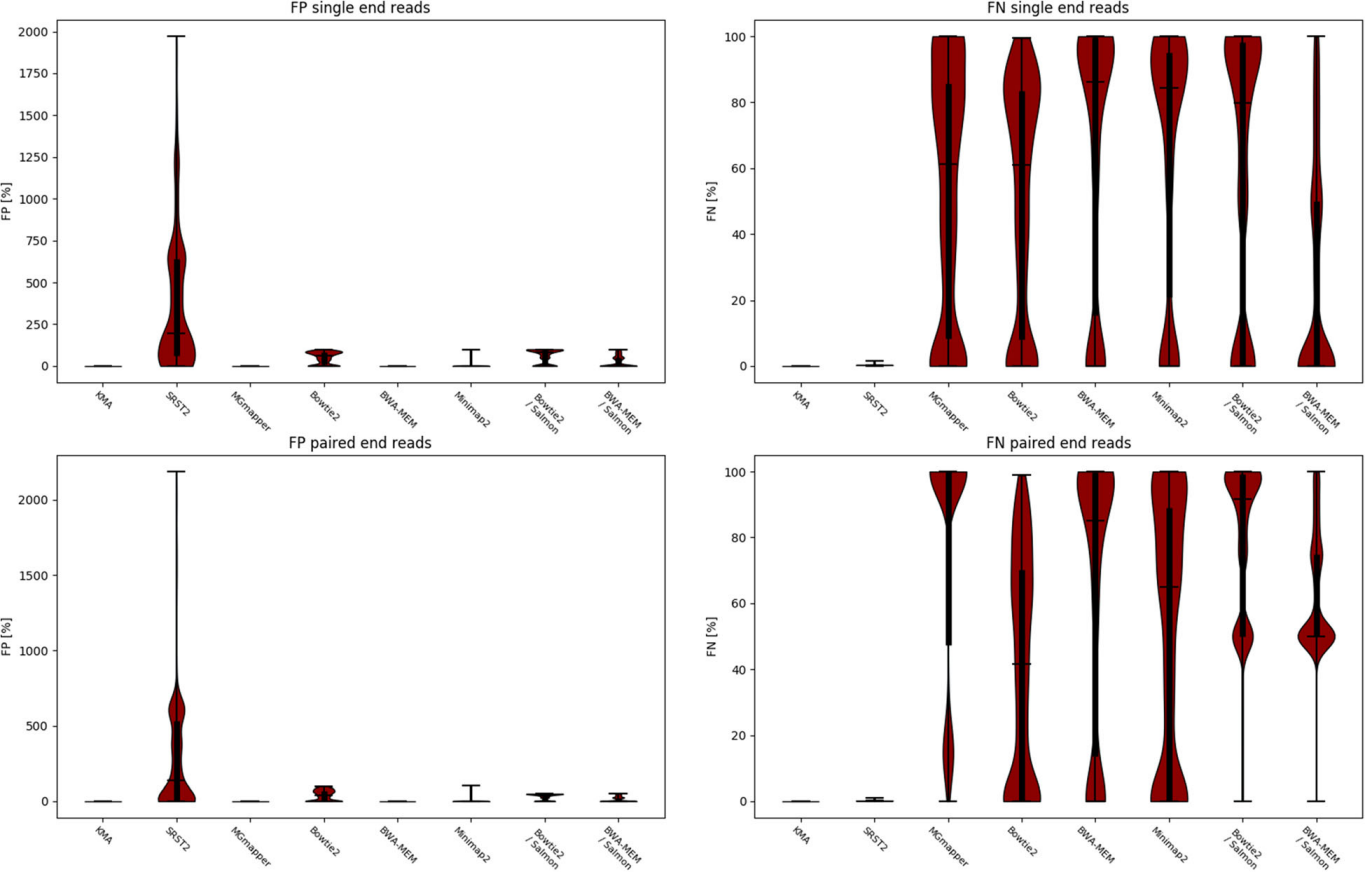
Distribution of false positives (FP) and false negatives (FN), for KMA, SRST2, MGmapper, Bowtie2, BWA-MEM, MiniMap2 and Bowtie2 and BWA-MEM post processed with Salmon when mapping simulated reads from the ResFinder database back to the ResFinder database. A minimum mapping quality of 1 was used to ensure reproducibility.

For both the single and paired-end datasets, KMA mapped everything correctly, where every read in each sample was mapped to the correct template only (see Table 1). For all samples, SRST2 mapped almost all reads to the correct template, only differing by a read in most cases. However, since SRST2 allows for one read to map towards several templates and even several places within each template, a large number of false positives were achieved, thus giving a lower correlation score (see Table 1). For a few genes, SRST2 accepted up to 21 alignments (see Fig. 2). MGmapper did not assign any false positives, but underestimated the depth of the correct templates. This was especially the case for the paired-end dataset where MGmapper seemingly had trouble with coupling of the reads. This resulted in MGmapper only being able to assign 19.6% of the actual reads. BWA-MEM and MiniMap2 follow a similar pattern compared with MGmapper, where the methods are driven towards false negatives (FN). They do however perform better on the paired end reads. In contrast to the other methods tested, Bowtie2 has a more equal distribution between false positives (FP) and FN. Only BWA-MEM had improved predictions when post processed with Salmon, but only fro the single end reads, whereas Bowtie2 had a performance decrease, on both the single and paired end datasets (see Table 1 and Fig. 2).

**Table 1:** Performance of KMA, SRST2, MGmapper, BWA-MEM, Bowtie2, Minimap2 and Salmon, on simulated data generated from the ResFinder database. A minimum mapping quality of 1 was used to ensure reproducibility

Method / Performance	Single end read set			Paired end read set		
	MCC	Sensitivity	PPV	MCC	Sensitivity	PPV
KMA	1.000	1.000	1.000	1.000	1.000	1.000
SRST2	0.591	0.999	0.350	0.659	0.999	0.436
MGmapper	0.676	0.457	1.000	0.443	0.196	1.000
BWA-MEM	0.585	0.342	1.000	0.580	0.337	1.000
Bowtie2	0.480	0.480	0.480	0.577	0.577	0.577
Minimap2	0.591	0.353	0.988	0.671	0.455	0.991
BWA-MEM / Salmon	0.720	0.720	0.720	0.500	0.353	0.707
Bowtie2 / Salmon	0.390	0.389	0.398	0.250	0.177	0.368

*MCC* Matthews correlation coefficient*, PPV* Positive Prediction Value

### German / French *E. coli* outbreak

Ninety percent template coverage, 90% template identity and a minimum depth of 5 were the thresholds for predicting genes with KMA and SRST2. The identity threshold was skipped for MGmapper and methods post processed by SAMtools and BEDTools because introducing an identity threshold would require further post-processing, while only giving lower correlation scores. Only the depth threshold was used for Salmon, because Salmon only estimates the transcript levels of each template. Meaning that the exact location of each read is lost, making the analysis of template coverage and identity impossible.

MGmapper crashed when mapping the paired end reads, but succeeded in single end mode when only the forward reads were used. Paired end reads were converted to interleaved format for BWA-MEM because it crashed when the reads were split into two files.

### Predicting antimicrobial resistance

Full concordance between phenotypic susceptibility tests and the predicted antimicrobial resistance genes were achieved for both KMA and SRST2. Results for nalidixic acid resistance were excluded, since it is caused by chromosomal resistance genes that were not included in this study [16]. The only disagreement between KMA and SRST2 for this dataset were that SRST2 predicted the presence of dfrA17 and dfrA32 in addition to the dfrA7 gene predicted by KMA, in all of the outbreak strains (see Additional file 1: Table S1). A closer examination of this result revealed that the two additional genes predicted by SRST2 were due to multi mapping reads between them and dfrA7.

As for the simulated dataset, MGmapper had no FP predictions, but it missed most of the phenotypes. Even though the outbreak strains are thought to be highly similar, the results from MGmapper showed a highly diverse pattern between the samples (see Additional file 1: Table S1). Neither BWA-MEM, Bowtie2 nor Minimap2 predicted any genes exceeding the thresholds, no matter the settings of SAMtools and BEDTools (see Additional file 1: Table S2).

Better results were achieved for BWA-MEM and Bowtie2 when using Salmon, raising their correlation coefficient to 0.828 and 0.623, respectively (see Table 2). As for SRST2, the post-processing with Salmon gave several different hits for each allele, giving a bias towards FP. BWA-MEM combined with Salmon gave the same results for all outbreak strains, with no false negatives. A single false positive did however show up in all of the outbreak strains, the catB4 gene conferring resistance to chloramphenicol, which were tested phenotypically determined as susceptible. No clear pattern was observed for Bowtie2 combined with Salmon, where the predicted genes showed a diverse pattern across all outbreak strains.

**Table 2:** Performance measures of KMA, SRST2, MGmapper, BWA-MEM, Bowtie2, Minimap2 and Salmon, for predicting genes directly from raw reads. Thresholds for predicting a gene has been set to: 90% coverage, 90% identity and a minimum depth of 5. A minimum mapping quality of 10 was used for methods relying on post processing with SAMtools and BEDTools, as this gave the best performance across the tested thresholds

Mapping method	Post- processing method	Avg. mapping CPU time	Avg. post- processing CPU time	Peak memory	MCC
Predicting antimicrobial resistance					
KMA	NA	00:00:24.6	NA	42.3 MB	1.000
SRST2	NA	00:10:21.3	NA	165.0 MB	1.000
MGmapper^a^	NA	00:13:14.2	NA	101.4 MB	0.288
BWA-MEM	SAMtools / BEDTools	00:07:35.5	00:00:06.1	113.0 MB	0.000
BWA-MEM^b^	Salmon	00:07:34.5	00:00:12.2	694.9 MB	0.828
Bowtie2	SAMtools / BEDTools	00:02:35.5	00:00:06.7	33.7 MB	0.000
Bowtie2^b^	Salmon	00:03:16.4	00:02:24.5	935.8 MB	0.623
Minimap2	SAMtools / BEDTools	00:02:18.6	00:00:06.0	517.3 MB	0.000
Mapping towards cgMLST alleles					
KMA	NA	00:07:02.1	NA	8.3 GB	0.998
SRST2	NA	> 99:99:99.9	NA	NA	NA
MGmapper^a^	NA	01:23:21.5	NA	8.7 GB	0.062
BWA-MEM	SAMtools / BEDTools	02:14:50.8	00:14:06.7	8.9 GB	0.021
BWA-MEM^b^	Salmon	03:22:45.4	04:41:09.6	104.2 GB^P^	0.530
Bowtie2	SAMtools / BEDTools	01:50:56.8	00:15:23.5	4.1 GB	0.035
Bowtie2^b^	Salmon	> 99:99:99.9	NA	NA	NA
Minimap2	SAMtools / BEDTools	01:20:56.2	00:13:11.7	33.6 GB	0.035

^a^ MGmapper was executed on the forward reads only, as paired end mode crashed

^b^ Report all alignments

^P^ The post processing method was responsible for the peak memory consumption

### Mapping towards cgMLST alleles

The performance of each method was measured as their ability to find one allele within each locus of the cgMLST database, which together make the core genome of the bacteria.

As for the antimicrobial resistance genes, BWA-MEM, Bowtie2 and Minimap2 had trouble assigning genes exceeding the thresholds, thus giving a low correlation coefficient (see Table 2). The performance of MGmapper was slightly better than that of BWA-MEM used directly, giving a correlation of 0.062 compared with BWA-MEM with a correlation of 0.021 (see Table 2). As for the resistance gene prediction, the correlation of BWA-MEM was improved when using Salmon. In this case, however, Salmon did take up the majority of the computational resources when compared with the mapping. When estimating the allele abundances, Salmon had a peak memory consumption of 104.2 GB, and used more time on the post-processing than BWA-MEM used on mapping (see Table 2). KMA outperformed all of the methods with a correlation of 0.998, only missing two genes and detecting three duplicates on average.

SRST2 and Bowtie2 combined with Salmon were stopped after 100 h, where neither method had completed a single sample.

### Runtime analysis

CPU time and peak memory requirements were measured with GNU time (v1.7), mapping and post-processing were measured separately for all methods, unless included in the method. KMA outperformed all of the existing methods on speed when mapping both resistance genes and cgMLST genes. The memory usage of KMA was above that of Bowtie2, but just below that of MGmapper and BWA-MEM, and less than a fourth of MiniMap2 (see Table 2). The memory consumption and speed of KMA is thus feasible to carry out on a laptop, with results accurate enough to be used in surveillance.

## Discussion

BWA-MEM, Bowtie2 and Minimap2 were not designed to map against redundant databases, as they are based on unique mappings and therefore do not deal with multi mapping reads. This is also reflected by MGmapper, albeit relying on only one unique mapping.

Salmon was designed to predict RNA transcript levels, which includes a certain level of redundancy. The expression pattern seen in bacteria differs from that of eukaryotes, which Salmon was designed for, explaining some of the variation seen here. Salmon does, however, improve the predictions when used with BWA-MEM, with the major problem being over prediction, resulting in a number of FPs. Even though Salmon provides better predictions, it is hard to control the output because alignments are not included. This makes it difficult to sort out partial mapping templates, which might have improved the predictions.

SRST2 uses clustering of the template database in order to minimize the redundancy problem. It then uses Bowtie2 to map against the clusters and accepts all mappings, meaning that a single read can be assigned to several clusters. This is where SRST2 has its major drawback, as the clustering does not reflect the mapping performed afterwards. As recommended by SRST2, the databases were clustered with a 90% similarity threshold using CD-hit [6, 14]. However, two sequences being less than 90% identical can still share large stretches of identical DNA, opening the possibility for ties on a best matching template, which reduces the PPV for SRST2. Clustering based on identity will thus not give a non-redundant database, but rather make a stochastic limitation of the redundancy. This clustering of SRST2 may therefore be solved by clustering sequences together with long identical stretches of DNA, where the length of the stretch should conform to the read length used to map afterwards. When predicting phenotypic susceptibility, this feature of SRST2 is of less importance, as long as SRST2 finds at least one allele for each of the resistance genes present.

KMA was designed as a specialized solution for mapping against redundant databases, such as those seen in genomic epidemiology, and is therefore not a general solution compared with BWA-MEM, Bowtie2 and Minimap2. The memory usage of the index used with KMA is not linear, and is dependent on the redundancy within a given database. The higher the redundancy the slower the increase is in database size, whereas for non-redundant databases the indexing will grow faster than for BWA-MEM and Bowtie2.

BWA-MEM, Bowtie2 and Minimap2 have a large variety of parameters to set, but none of the parameters seem to have a dramatic effect on the results achieved here.

The ConClave scoring scheme is the major difference between KMA and the methods compared, with respect to the prediction performance. If the ConClave scoring had been adapted to analyze SAM-format, BWA-MEM and Bowtie2 would probably have had a similar prediction performance compared with that of KMA. The speed advantage of KMA lies mainly in the heuristic *k*-mer mapping, which makes KMA able to discard non-mapping sequences at an early state or at least limit the search space.

## Conclusion

Alignment of sequences plays a crucial role in bioinformatics, and is often the cornerstone in sequence analysis. Choosing the right alignment method for the right problem is thus evident for the further analysis. For aligning raw reads directly against redundant databases, KMA outperforms existing methods on both speed, precision and sensitivity.

## Additional file

Additional file 1: Table S1 includes a detailed overview of the predicted resistance genes from each sample, using the methods described in this study. Table S2 extends Table 2, by measuring Matthews correlation coefficient under different thresholds on mapping quality and whether the reads are properly paired. (DOCX 79 kb)

## Abbreviations

FN: False negative
FP: False positive
MCC: Matthews correlation coefficient
MLST: Multi locus sequence type
PPV: Positive predictive value
TN: True negative
TP: True positive
