# Supplementary material for: Rapid and precise alignment of raw reads against redundant databases with KMA
Source: BMC Bioinformatics. 2018 Aug 29;19:307. doi: 10.1186/s12859-018-2336-6 (PMC6116485; doi:10.1186/s12859-018-2336-6)
Supplement: Supplementary file 1 — Table S1 includes a detailed overview of the predicted resistance genes from each sample, using the methods described in this study. Table S2 extends Table 2, by measuring Matthews correlation coefficient under different thresholds on mapping quality and whether the reads are properly paired. (DOCX 79 kb) [file 12859_2018_2336_MOESM1_ESM.docx]

| Method / Gene | KMA | SRST2 | MGmapper | Bowtie2 / Salmon | BWA-MEM / Samon |
| --- | --- | --- | --- | --- | --- |
| strA | A | A | Ec11-4404 | C236-11^3a^,  C227-11,  Ec11-3677,  Ec11-4404^2a^, Ec11-4522^5a^, Ec11-4623^5a^, Ec11-4632-C1^5a^, Ec11-4632-C2^3a^, Ec11-4632-C3^5a^, Ec11-4632-C4^5a^, Ec11-4632-C5 | C236-11^3a^, C227-11^4a^,  Ec11-3677^4a^, Ec11-4404^5a^, Ec11-4522^5a^, Ec11-4623^5a^, Ec11-4632^C, 5a^ |
| strB | A | A | A | C236-11^2a^,  C227-11,  Ec11-3677,  Ec11-4404,  Ec11-4522,  Ec11-4623,  Ec11-4632^C^ | C236-11^2a^, C227-11,  Ec11-3677^2a^, Ec11-4404^2a^, Ec11-4522^2a^, Ec11-4623^2a^,  Ec11-4632^C, 2a^ |
| aadA1 | - | - | - | A | A |
| blaCTX-M-15* | A | A | - | C236-11^2a^,  Ec11-3677^2a^ | A |
| blaCTX-M-X** | - | - | - | C236-11^2v^,  Ec11-3677,  Ex11-4404 | - |
| blaTEM-1B | A | A | - | C236-11,  C227-11,  Ec11-3677,  Ec11-4404,  Ec11-4623,  Ec11-4632-C2 | A |
| blaTEM-X*** | - | - | - | C236-11^7v^,  C227-11^4v^,  Ec11-3677^2v^, Ec11-4404^10v^, Ec11-4623^16v^, Ec11-4632-C2^20v^, Ec11-4632-C3 | - |
| sul1 | A | A | - | C236-11^2a^,  C227-11^2a^,  Ec11-3677,  Ec11-4404^2a^, Ec11-4522^2a^, Ec11-4623^2a^,  Ec11-4632^C, 2a^ | A |
| sul2 | A | A | C236-11,  Ec11-4404, Ec11-4623, Ec11-4632-C1, Ec11-4632-C2, Ec11-4632-C3, Ec11-4632-C4 | C236-11^2a^, C227-11,  Ec11-3677^2a^, Ec11-4404^4a^, Ec11-4522^2a^, Ec11-4623^3a^, Ec11-4632-C1^2a^, Ec11-4632-C2^3a^, Ec11-4632-C3^2a^, Ec11-4632-C4^3a^, Ec11-4632-C5 | C236-11^2a^, C227-11^2a^,  Ec11-3677^3a^, Ec11-4404^4a^, Ec11-4522^3a^, Ec11-4623^4a^, Ec11-4632-C1^4a^, Ec11-4632-C2^3a^, Ec11-4632-C3^3a^, Ec11-4632-C4^3a^, Ec11-4632-C5^3a^ |
| tet(A) | A | A | - | C236-11^2a^,  C227-11,  Ec11-3677,  Ec11-4404^3a^, Ec11-4522^3a^, Ec11-4623^4a^, Ec11-4632-C1^3a^, Ec11-4632-C2^3a^, Ec11-4632-C3^3a^, Ec11-4632-C4^5a^, Ec11-4632-C5^3a^ | A^3a^ |
| dfrA7 | A | A | A | C236-11^2a^,  C227-11^2a^,  Ec11-3677^2a^, Ec11-4404^2a^, Ec11-4522^2a^, Ec11-4623^2a^, Ec11-4632-C1^2a^, Ec11-4632-C2^2a^, Ec11-4632-C3, Ec11-4632-C4^2a^, Ec11-4632-C5 | A^2a^ |
| dfrA16 | - | - | - | - | A |
| dfrA17 | - | A | - | - | - |
| dfrA32 | - | A | - | - | - |
| catB4 | - | - | - | A | A |

Table S1; Overview of which genes were found in which samples, for the German / French E. coli outbreak and two historical E. coli’s (Ec04-8351 and Ec09-7901). *: Conventionally verified using PCR, A: All outbreak strains: Ec11-3677, Ec11-4404, Ec11-4522, Ec11-4623, Ec11-4632^C^, C227-11 & C236-11. **: Different variants detected, blaCTX-M-15 excluded. ***: Different variants detected, blaTEM-1B excluded. ^C^: Clone samples C1-C5. ^Xa^: X different alleles found. ^Xv^: X different variants found.

| Database | Mapping method | Post- processing method | Avg. mapping  CPU time | Avg. post- processing CPU time | Peak memory | MCC |
| --- | --- | --- | --- | --- | --- | --- |
| Predicting antimicrobial resistance | | | | | | |
|  | KMA | NA | 00:00:24.6 | NA | 42.3 MB | 1.000 |
|  | SRST2 | NA | 00:10:21.3 | NA | 165.0 MB | 1.000 |
|  | MGmapper^#^ | NA | 00:13:14.2 | NA | 101.4 MB | 0.288 |
|  | BWA-MEM | Samtools / bedtools^q10^ | 00:07:35.5 | 00:00:06.1 | 113.0 MB | 0.000 |
|  | BWA-MEM | Samtools / bedtools^q10,pp^ | 00:07:35.5 | 00:00:05.9 | 113.0 MB | 0.000 |
|  | BWA-MEM | Samtools / bedtools^q20^ | 00:07:35.5 | 00:00:06.0 | 113.0 MB | 0.000 |
|  | BWA-MEM | Samtools / bedtools^q20,pp^ | 00:07:35.5 | 00:00:05.9 | 113.0 MB | 0.000 |
|  | BWA-MEM | Samtools / bedtools^q30^ | 00:07:35.5 | 00:00:06.0 | 113.0 MB | 0.000 |
|  | BWA-MEM | Samtools / bedtools^q30,pp^ | 00:07:35.5 | 00:00:05.9 | 113.0 MB | 0.000 |
|  | BWA-MEM* | Salmon | 00:07:34.5 | 00:00:12.2 | 694.9 MB | 0.828 |
|  | Bowtie2 | Samtools / bedtools^q10^ | 00:02:35.5 | 00:00:06.7 | 33.7 MB | 0.000 |
|  | Bowtie2 | Samtools / bedtools^q10,pp^ | 00:02:35.5 | 00:00:06.6 | 33.7 MB | 0.000 |
|  | Bowtie2 | Samtools / bedtools^q20^ | 00:02:35.5 | 00:00:06.7 | 33.7 MB | 0.000 |
|  | Bowtie2 | Samtools / bedtools^q20,pp^ | 00:02:35.5 | 00:00:06.5 | 33.7 MB | 0.000 |
|  | Bowtie2 | Samtools / bedtools^q30^ | 00:02:35.5 | 00:00:06.5 | 33.7 MB | 0.000 |
|  | Bowtie2 | Samtools / bedtools^q30,pp^ | 00:02:35.5 | 00:00:06.4 | 33.7 MB | 0.000 |
|  | Bowtie2* | Salmon | 00:03:16.4 | 00:02:24.5 | 935.8 MB | 0.623 |
|  | Minimap2 | Samtools / bedtools^q10^ | 00:02:18.6 | 00:00:06.0 | 517.3 MB | 0.000 |
|  | Minimap2 | Samtools / bedtools^q10,pp^ | 00:02:18.6 | 00:00:05.8 | 517.3 MB | 0.000 |
|  | Minimap2 | Samtools / bedtools^q20^ | 00:02:18.6 | 00:00:05.9 | 517.3 MB | 0.000 |
|  | Minimap2 | Samtools / bedtools^q20,pp^ | 00:02:18.6 | 00:00:05.8 | 517.3 MB | 0.000 |
|  | Minimap2 | Samtools / bedtools^q30^ | 00:02:18.6 | 00:00:05.8 | 517.3 MB | 0.000 |
|  | Minimap2 | Samtools / bedtools^q30,pp^ | 00:02:18.6 | 00:00:05.8 | 517.3 MB | 0.000 |
| Mapping towards cgMLST alleles | | | | | | |
|  | KMA | NA | 00:07:02.1 | NA | 8.3 GB | 0.998 |
|  | SRST2 | NA | >99:99:99.9 | NA | NA | NA |
|  | MGmapper^#^ | NA | 01:23:21.5 | NA | 8.7 GB | 0.062 |
|  | BWA-MEM | Samtools / bedtools^q10^ | 02:14:50.8 | 00:14:06.7 | 8.9 GB | 0.021 |
|  | BWA-MEM | Samtools / bedtools^q10,pp^ | 02:14:50.8 | 00:15:22.8 | 8.9 GB | 0.000 |
|  | BWA-MEM | Samtools / bedtools^q20^ | 02:14:50.8 | 00:14:05.6 | 8.9 GB | 0.020 |
|  | BWA-MEM | Samtools / bedtools^q20,pp^ | 02:14:50.8 | 00:15:09.0 | 8.9 GB | 0.000 |
|  | BWA-MEM | Samtools / bedtools^q30^ | 02:14:50.8 | 00:14:20.9 | 8.9 GB | 0.020 |
|  | BWA-MEM | Samtools / bedtools^q30,pp^ | 02:14:50.8 | 00:15:10.8 | 8.9 GB | 0.000 |
|  | BWA-MEM* | Salmon | 03:22:45.4 | 04:41:09.6 | 104.2 GB^P^ | 0.530 |
|  | Bowtie2 | Samtools / bedtools^q10^ | 01:50:56.8 | 00:15:23.5 | 4.1 GB | 0.035 |
|  | Bowtie2 | Samtools / bedtools^q10,pp^ | 01:50:56.8 | 00:15:18.1 | 4.1 GB | 0.033 |
|  | Bowtie2 | Samtools / bedtools^q20^ | 01:50:56.8 | 00:15:18.0 | 4.1 GB | 0.033 |
|  | Bowtie2 | Samtools / bedtools^q20,pp^ | 01:50:56.8 | 00:15:23.0 | 4.1 GB | 0.032 |
|  | Bowtie2 | Samtools / bedtools^q30^ | 01:50:56.8 | 00:15:12.3 | 4.1 GB | 0.033 |
|  | Bowtie2 | Samtools / bedtools^q30,pp^ | 01:50:56.8 | 00:15:36.7 | 4.1 GB | 0.029 |
|  | Bowtie2* | Salmon | >99:99:99.9 | NA | NA | NA |
|  | Minimap2 | Samtools / bedtools^q10^ | 01:20:56.2 | 00:13:11.7 | 33.6 GB | 0.035 |
|  | Minimap2 | Samtools / bedtools^q10,pp^ | 01:20:56.2 | 00:14:22.3 | 33.6 GB | 0.000 |
|  | Minimap2 | Samtools / bedtools^q20^ | 01:20:56.2 | 00:13:18.5 | 33.6 GB | 0.035 |
|  | Minimap2 | Samtools / bedtools^q20,pp^ | 01:20:56.2 | 00:14:20.1 | 33.6 GB | 0.000 |
|  | Minimap2 | Samtools / bedtools^q30^ | 01:20:56.2 | 00:13:18.1 | 33.6 GB | 0.028 |
|  | Minimap2 | Samtools / bedtools^q30,pp^ | 01:20:56.2 | 00:14:17:1 | 33.6 GB | 0.000 |

Table S2; Performance measures of KMA, SRST2, MGmapper, BWA-MEM, Bowti2, Minimap2 and Salmon, for predicting genes directly from raw reads. Thresholds for predicting a gene has been set to: 90% coverage, 90% identity and a minimum depth of 5. #: MGmapper was executed on the forward reads only, as paired end mode crashed. *: Report all alignments. qX: Accept all alignments with a mapping quality above X. pp: Accept only properly paired reads. P: post-processing method had the peak memory usage.
